# The viral landscape in metastatic solid cancers

**DOI:** 10.1016/j.heliyon.2025.e42548

**Published:** 2025-02-08

**Authors:** Robin Mjelle, Ícaro Castro, Kristin Roseth Aass

**Affiliations:** a Department of Cancer and Molecular Medicine, Norwegian University of Science and Technology, Trondheim, Norway; b Department of Pathology, St.Olavs Hospital, Trondheim, Norway; c University of Sao Paulo, Brazil

## Abstract

Here, we analyze the viral landscape in blood and tissue from 4918 metastatic cancer patients across 38 solid cancer types from the Hartwig Medical Foundation (HMF) cohort, the largest pan-cancer study on metastatic cancer. Using a coverage-based filtering approach, we detected 25 unique viral genera across 32 different cancer types, with a total of 747 unique virus-positive tissue samples. We detected 336 virus-positive blood samples across 29 cancer types, dominated by *Torque teno virus* and *Alphatorquevirus*. The tissue samples were dominated by *Alphapapillomavirus* and *Roseolovirus*. *Alphapapillomavirus* was significantly enriched in genital, anal, and colorectal cancers and was associated with host mutational signatures and transcriptional programs related to immunity and DNA repair. Host genes with *Alphapapillomavirus* integration tended to be more highly expressed and samples with HPV integration had higher somatic mutation rates and higher number of extrachromosomal DNA elements. *Alphapapillomavirus* was also detected in a significant proportion of blood samples from cervix and anal cancers, suggesting a potential blood-based biomarker.

## 1 Introduction

Several viral species have been shown to be associated with cancer and virus infection is estimated to be the central cause of about 10 % of cancer cases [1]. The best-known example is the association between Human papillomavirus (HPV) and genital cancer, head and neck cancer and anal cancer [2]. Other associations include Epstein-Barr virus (EBV) with different lymphomas and nasopharyngeal carcinoma, Hepatitis B virus (HBV) with hepatocellular carcinoma, and human T-cell lymphotropic virus type 1 with adult T-cell leukemia [3]. The molecular mechanisms of virus-induced carcinogenesis vary between virus types; however, interactions between viral oncoproteins and host tumor suppressor proteins are fundamental for DNA viruses [4]. The viral landscape has been characterized in other pan-cancer datasets from primary cancer tissue, demonstrating that viruses such as Lymphocryptovirus (EBV), *Orthohepadnavirus* (HBV), *Roseolovirus* (HHV) and *Alphapapillomavirus* (HPV) are present across cancer types [5,6]. A few studies have analyzed the presence of viruses in metastatic tissue, focusing on well-known oncogenic viruses. Using targeted methods such as real-time qPCR and Western blots, HPV and EBV have been detected in metastatic tissue from primary squamous cell carcinoma and nasopharyngeal carcinoma, while Cytomegalovirus (HCMV) has been detected in brain metastases from colorectal and breast cancers [7,8]. However, knowledge about the viral landscape in metastatic tumors across different cancer types remains limited, likely due to the inability of traditional analytical methods to perform comprehensive viral profiling and because metastatic samples have not been included in pan-cancer sequencing studies such as The Cancer Genome Atlas (TCGA) and Pan-Cancer Analysis of Whole Genomes (PCAWG) [5,6]. In this study we analyze the presence of virus within the metastatic tissue and matched blood samples of the Hartwig Medical Foundation (HMF)-cohort, the largest pan-cancer study on metastatic cancer [9]. This dataset contains whole-genome sequencing (WGS) data derived from biopsies of 38 different cancer types and therefore represents a unique opportunity to perform a pan-cancer characterization of the virome in a broad range of metastatic tissue.

Here, we present the viral landscape of metastatic tumors based on alignment- and coverage-based filtering analysis of 4952 WGS samples from the HMF cohort. Additionally, we have analyzed WGS data from matched blood samples for all patients and RNA-seq data from tumor samples from over half of the patients.

## 2 Results

### 2.1 Identification of virus in tumor and blood of metastatic solid cancer patients

**Fig. 1:**
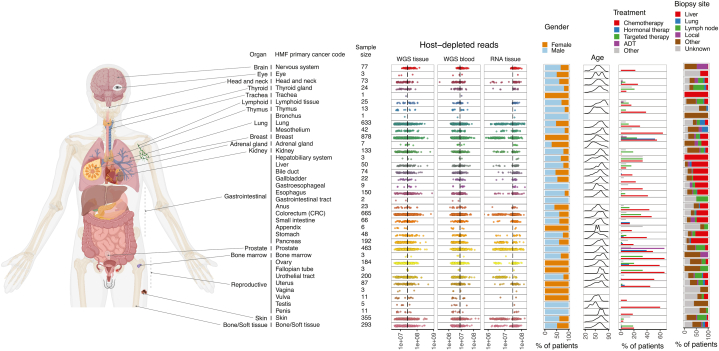
**Overview of anatomic location and clinicopathological factors of the HMF cohort.** Anatomic location of the 38 cancer types analyzed in the current study. The cancer types are ordered based on the anatomical location. Host-depleted reads indicate the number of reads used as input for the virus analyses. Treatment indicates pre-biopsy treatment and includes cases where patients received multiple treatments. Biopsy site indicates the anatomic location of the biopsy collection-site.

We examined the viral landscape of the Hartwig Medical Foundation (HMF) cohort [9], a metastatic pan-cancer resource comprising 4918 patients from 38 different cancer types (Fig. 1). We analyzed 4952 whole-genome sequencing (WGS) tissue samples, 4902 WGS blood samples and 2544 RNA-sequencing (RNA-seq) tissue samples. Per sample, we analyzed, on average 27.4 million reads from WGS tissue, 30.3 million reads from RNA-seq tissue and 8.2 million reads from WGS blood (Fig. 1). About half of the patients (n = 2256) received treatment before biopsy collection, of which chemotherapy was the most common treatment type (Fig. 1). Information about biopsy location was available for 80 % of the patients, and of those, 6.6 % of the samples were collected from the primary tumor, 20 % from lymph nodes and 73.4 % from distant organs (Fig. 1). The ratio between host-depleted reads and total reads in the three datasets were relatively similar between the cancer types, with an average percentage of host-depleted reads of 1 % for WGS from tissue, 0.3 % for WGS from blood and 1.15 % for RNA-Seq from tissue (Figs. S1A–C).

**Fig. 2:**
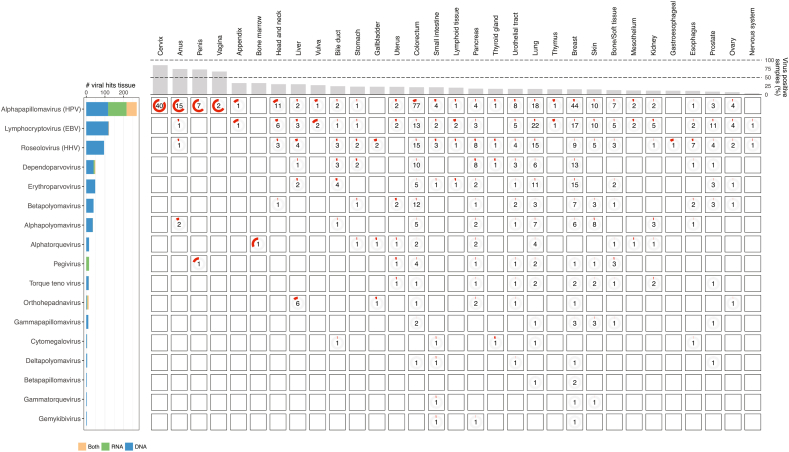
**Overview of virus genera in metastatic cancer tissue.** Number of viruses detected in metastatic cancer tissue grouped by the primary cancer types. Shown are viruses detected in more than one sample. The numbers indicate the absolute number of virus-positive samples within the cancer type and the red circle indicates the percentage of virus-positive samples relative to the total number of samples within the cancer type. A sample that was positive for at least one viral species within the specific genus was regarded as virus-positive for that genus. The left bar-plot shows the total number of viral-positive samples across all cancer types coloured by which method was used to detect the virus. The top bar-plot shows the percentage of virus-positive samples for each primary cancer type when including all virus types.

Viruses were identified through direct alignments to human-associated viral species from the NCBI RefSeq viral database, followed by a coverage-based filtering approach to remove low-abundant and potential false-positive hits. Viruses were considered present in a sample only if they had a minimum genome coverage of at least 10 % (see Methods). Further, only viruses that are known to be part of the human microbiome were included in the analysis. Using a coverage-based filtering approach, we detected 25 unique viral genera across 32 different cancer types, and a total of 747 (15 %) unique virus-positive tumor samples when including data from both DNA and RNA (Fig. 2, Fig. S1D). The top-three most prevalent taxa were HPV, EBV and HHV, comprising almost 70 % of the viral hits (Fig. 2). When combining the genome coverage data from virus-positive samples, we obtained complete genome coverage for most of the viruses at the DNA-level, and HPV, EBV, HBV, and pegivirus showed complete coverage at the RNA-level (Figs. S2A–B). This indicates that both DNA and RNA-based methods can be used to detect viruses in tissue samples. Indeed, for the most prevalent taxa, HPV and EBV, we found significant correlation between the read counts in the WGS and RNA-seq data, whereas for HHV, the correlation was weaker but still significant (Fig. S2C).

The tumor samples in the HMF cohort were sequenced with an average of 2.6 billion reads per sample. To assess whether lower sequencing depths could still enable virus detection, we performed virus profiling using 100 %, 75 %, and 50 % of the WGS data and calculated the percentage of virus-positive samples using a 10 % coverage threshold. As expected, the fraction of virus-positive samples decreased with fewer input reads (Fig. S2D). However, while EBV and HHV showed slightly greater reductions of virus-positive samples when using 50 % of input reads (31 % and 19 % reduction, respectively), the detection rate for HPV declined by just 10 %. Thus, sequencing depth will influence the sensitivity of viral detection, but highly expressed viruses, such as HPV would still be detected in lower depth sequencing samples, suggesting that WGS could have diagnostic value for viral profiling.

Looking further into the viral landscape beyond the most prevalent taxa, some viruses with known human associations were detected in single samples although with a genome-coverage above 10 %, including *alphainfluenzavirus* (seasonal flu), *betacoronavirus* [10], *betatorquevirus* [11], *bocaparvovirus* [12], *norovirus* (viral gastroenteritis), orthopneumovirus (respiratory syncytial virus), *pneumoviridae* and *protoparvovirus* [13]. Several additional viruses were detected with genome coverage between 1 and 10 %, both in RNA and DNA (Fig. S3A).

**Fig. 3:**
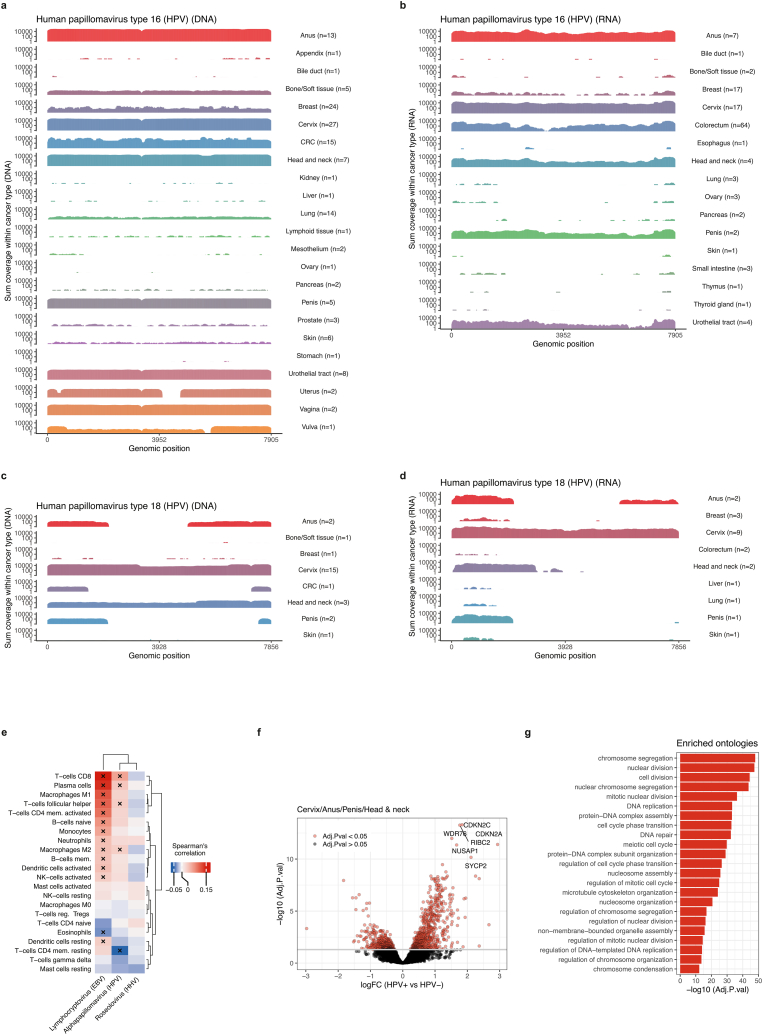
**Prevalence of human *alphapapillomavirus* A)** Summed genome coverage for HPV-16 in the DNA-data stratified by cancer type. Number of samples with >10 % genome coverage are indicated for each cancer type. Genomic position is shown on the x-axis. **B)** Similar as in A) for the RNA-seq data. **C)** Similar as in A) for the HPV-18 for DNA-seq data. **D)** Similar as in C) for RNA-seq data. **E)** Spearman's correlation between virus coverage estimates and immune cell types from CIBERSORTx. Unsupervised clustering is performed on the viruses and immune cells. Cross-sign indicates significant correlation (adjusted p-values <0.05). **F)** Volcano plot showing the differentially expressed genes between HPV+ and HPV- samples across the cancer types cervix, anus, penis and head and neck. The y-axis shows the -log10 inverted p-values and the x-axis the log2 fold-change between the groups. Red points indicate significant differentially expressed genes as detected by *limma-voom* after Benjamini-Hochberg p-value adjustment. The most significant genes are indicated with names. **G)** Enriched biological processes for genes up-regulated in HPV + samples in the four cancer types listed in F). The x-axis shows the -log10 inverted p-values after Benjamini-Hochberg adjustment. Shown are the top most significant biological processes.

We detected a significant enrichment of HPV in cervix, penis, anus and head and neck (p = 1.1e-15; p = 1.1e-15; p = 1.3e-14, p = 4.9e-4, respectively, Chi-squared test), consistent with current knowledge about the prevalence of HPV in these cancer types [[14], [15], [16]] (Fig. 2). Interestingly, we also find a significant enrichment of HPV in CRC (p = 2.1e-11, Chi-squared test), an association not previously observed in large pan-cancer datasets [5,6]. This association remains controversial, though individual studies have detected HPV in CRC primary tissue [17]. The most prevalent HPV-species, HPV-16 and HPV-18, showed high prevalence both at the RNA and DNA level for the abovementioned cancer types (Fig. 2, Fig. 3A–D). For HPV-16, both coverage width and coverage depth varied between cancer types, and cancer types with large group sizes such as prostate and breast did not show higher prevalence than the less common cancer types, such as penis and anus where you expect to find the virus, indicating that the presence of the virus reflects the biology of the organ of origin (Fig. 3A–D). HPV was detected in biopsies from several body sites (Fig. S3B), with highest frequency in liver and lymph nodes, indicating that HPV will spread with primary cancer cells and can be detected in multiple organs irrespectively of tissue trophism of HPV. This also indicates that HPV-positive metastatic biopsies are not a result of the metastases being located in typically HPV-positive regions, such as the head and neck or the reproductive organs. The most prevalent species was HPV-16 followed by HPV-18; however HPV-18 was restricted primarily to cancers of cervix, penis, anus and head and neck (Fig. 3C and D). HPV was also detected in several other cancer types (Fig. 2, Fig. 3A–D), which could potentially be explained by the general prevalence of HPV in the population independent of cancer diagnosis.

Since HPV was significantly enriched in cancer types known to be HPV-associated, we investigated whether HPV could help distinguish different cancer types based on primary tumor location. To assess this, we calculated the Area Under the Receiver Operating Characteristic curve (AUC-ROC) values for each cancer type against all others using two approaches: a binary classification with a 10 % HPV coverage threshold and continuous HPV coverage values as diagnostic markers. Our analysis revealed that cervix, penis, anus, and vaginal cancers were distinctly separated from other cancer types, each with an AUC above 0.8 (Fig. S3C). However, HPV alone was insufficient to differentiate these four cancer types from one another (Fig. S3D). These findings suggest that high HPV levels in metastases point towards a HPV associated cancer of origin, but additional biomarkers are needed for a more robust classification.

The herpesviruses EBV and HHV were detected across most cancer types. EBV was detected in more than 100 samples, although no specific enrichment within specific cancer types was found (Fig. 2, Figs. S4A–B). The coverage analysis showed high coverage of EBV within lymphoid tissue and head and neck cancers, in agreement with the known association between EBV and tumors originating from head and neck and lymphoid tissue [18](Fig. S4A). Interestingly, lymphoid and head and neck cancers were the only malignancies that showed expression of EBV at the RNA-level, indicating that EBV is present in both the latent and lytic phase in these cancers (Fig. S4B). We did not observe enrichment of EBV-positive samples from specific biopsy sites (Fig. S4C).

Given the established role of the immune system in responding to viral infections, we next investigated whether specific immune cell types were enriched or depleted in relation to the tumor's viral content. Epstein-Barr virus (EBV) is known to modulate the tumor microenvironment by inducing immune cell infiltration and altering immune cell pathways, such as induction of plasma, CD4 and CD8 T-cells [19,20]. In contrast, other viruses, such as human herpesviruses (HHVs), often establish latency with minimal immune activation [21]. To explore if similar immune interactions are present in metastatic tissue, we performed immune-cell estimation of the RNA-seq data using CIBERSORTx and correlated the virus coverage breadth values of the three most prevalent viruses with the estimated immune cell content. As expected, EBV showed significant positive correlation with several immune cell types, most significant being CD8 T-cells and plasma cells (Fig. 3E). HHV was also detected across several cancer types without any specific cancer-specific enrichment of the virus (Fig. S4D), and we did not find evidence of immune cell association (Fig. 3E). Further, we did not detect RNA-transcription from any of the HHV-species, confirming the known latent state of the virus [22].

Among the less prevalent taxa, we detected alphapolyomavirus (merkel cell polyomavirus isolate R17b) within three merkel cell carcinomas, in agreement with known etiology of skin cancer (Fig. 2). We obtained complete genome-coverage of the RNA virus pegivirus, with a total of 15 positive samples across nine cancer types, including penis cancer (Fig. 2 and Fig. S2B). We detected transcription of four different retroviruses, including betaretrovirus*,* deltaretrovirus, gammaretrovirus and human endogenous retrovirus, the latter which were expressed in all except 17 RNA-seq samples (Fig. S2B). HBV was detected in pancreas and liver cancer, of which 12 % (six samples) of liver cancer patients were HBV-positive, in agreement with the etiology of liver cancer [23] (Fig. 2). Of the HBV-positive liver samples, all had HBV in the RNA-data and five in the DNA-data.

### 2.2 Alphapapillomavirus is associated with specific host transcriptional programs and mutational signatures in metastases

HPV has u been shown to impact the host genome by activating and suppressing different transcriptional programs, methylation status and somatic mutations [6,[24], [25], [26], [27]]. Since viruses were detected in metastatic lesions across most cancer types, we focused on HPV, the most prevalent virus in our dataset. The metastases originating from cancers of cervix, anus, penis and head and neck showed the highest relative prevalence of HPV, and we therefore focused the analysis on these cancer types. We grouped samples from these cancer types into two groups: HPV-positive and HPV-negative samples, defined as samples with and without detected HPV. Utilizing the RNA-seq data we investigated if HPV-positive samples had a different gene expression profile than HPV-negative samples. We detected significant gene expression changes between the groups, with 660 upregulated genes and 398 downregulated genes (Fig. 3F–Supplementary Table S1). Among the significantly up-regulated genes we found an enrichment of genes related to cell-cycle and DNA-repair, in agreement with previous findings that HPV interferes with cell-cycle regulation and the DNA-repair machinery [28,29], supporting that these pathways are activated in the presence of HPV (Fig. 3G). As an example, CDKN2A and CDKN2C were among the most up-regulated genes in HPV-positive samples and are both involved in the p53/Rb pathway. The HPV E6 and E7 proteins are thought to induce tumor formation by interacting with the tumor suppressors p53 and pRB, respectively, likely explaining the dysregulation of the CDKN2-genes [30].

We observed a significant mutual exclusive association between TP53 driver mutation and the presence of HPV within cervix, anus, penis and head and neck, a phenomenon previously described in cervix and head and neck cancers in the PCAWG dataset [5] and mechanistically caused by HPV binding to TP53, leading to ubiquitin-mediated degradation of the protein [5,[31], [32], [33]] (Fig. S4E). Thus, HPV dependent TP53 degradation is also likely present in metastases across multiple cancer types.

**Fig. 4:**
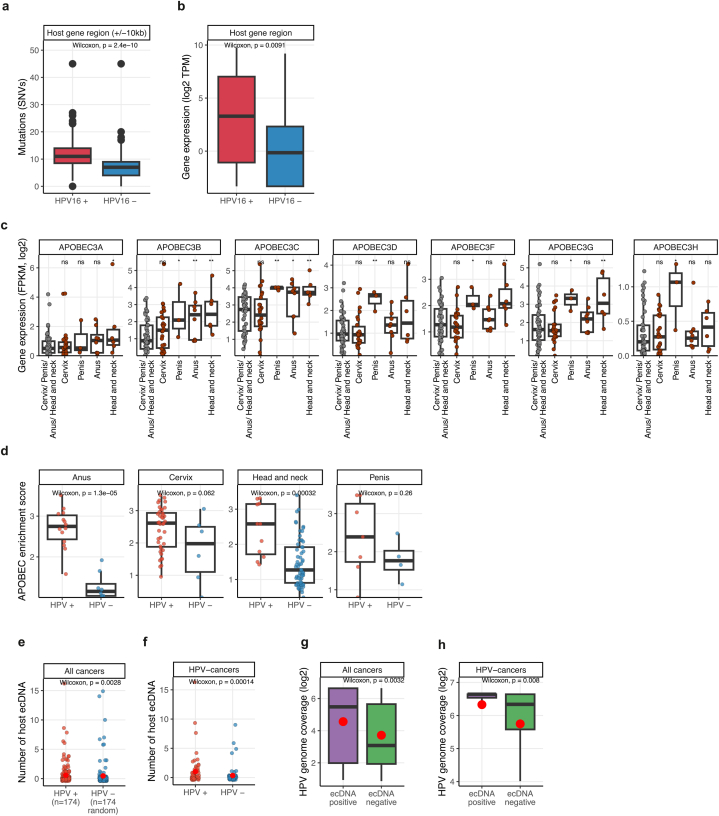
**HPV virus integration and gene expression A)** Boxplot showing number of single nucleotide variants (SNVs) for samples with detected HPV (HPV+) and samples without HPV (HPV-) for the cancer types cervix, anus, penis and head and neck. SNVs in a region of 20 kb around the integration sites are counted. The same genomic region is compared for HPV+ and HPV- samples. **B)** Boxplot showing the difference in host gene expression for samples with and without HPV for the cancer types cervix, anus, penis and head and neck. Compared is the expression of the same genes in samples with and without HPV-integration. **C)** Host gene expression of APOBEC genes in samples with HPV-integration (red points) and samples without HPV-integration (grey points). The p-values are the results of the comparison to the samples with no HPV-integration (two-sided Wilcoxson test, BH-adjusted). **D)** APOBEC enrichment score as estimated from the *MutationalPatterns* R package of HPV+ and HPV- samples in cancer types cervix, anus, penis and head and neck. P-values from a two-sided Wilcoxon test between the groups. **E)** Number of ecDNA for HPV + samples and a random set of HPV- samples. All cancer types were considered both for the HPV+ and HPV- samples. The p-value is from a two-sided Wilcoxon test. Mean values are indicated with a red dot **F)** Number of ecDNA for HPV + samples and HPV- samples for the cancer types cervix, anus, penis and head and neck. The p-value is from a two-sided Wilcoxon test. Mean values are indicated with a red dot **G)** HPV genome coverage values for ecDNA positive samples and ecDNA negative samples when considering all cancer types. The p-value is from a two-sided Wilcoxon test. Mean values are indicated with a red dot **H)** Similar as in G) for the cancer types cervix, anus, penis and head and neck. The p-value is from a two-sided Wilcoxon test. Mean values are indicated with a red dot.

Viral integration into the host genome can cause significant changes in the cell [34]. Integration of viral DNA into the human genome was investigated using VirusBreakend [35], a tool that uses a custom Kraken2 database [36] to classify the virus. In total, 1118 viral integration events were detected across 13 different viral species. The highest number of viral events were found for EBV (HHV-4) and HPV (HPV−16) with 491 and 419 integration events, respectively (Fig. S5A). All chromosomes contained integrations, and most integrations occurred in intergenic and intronic regions, but 47 integrations were found within exons (Fig. S5B). Cervix cancer had the highest number of integration events, most of which (76 %) were integration of HPV-16 (n = 162) and HPV-18 (n = 22) (Fig. S5C). 35 of the integration events occurred within exons of protein coding genes, including known oncogenes such as ERBB2 and transcription factors such as FOXA3 (Fig. S5D). Focusing on the four cancers with enrichment of HPV-16 integrations, cervix, anus, penis and head and neck, we found that HPV-16 positive samples had significant higher number of single nucleotide variants (SNVs) within a 20 kb region surrounding integration sites within gene bodies (p = 2.5e^−10^, two-sided Wilcoxon test - compared to the number of SNV within the same genomic regions in a random set samples lacking HPV-16 integrations) (Fig. 4A). Next, for the same four cancer types, we found that genes with detected HPV-16 integration had higher expression in integration-positive samples compared to integration-negative samples (p = 0.0091, two-sided Wilcoxon test), indicating that HPV has a positive effect on gene-expression upon integration within the gene region (Fig. 4B). Having shown that the number of SNVs differed between HPV-16 positive samples and HPV-16 negative samples, we analyzed copy number variants using the same approached as described for SNVs, focusing on the four cancers with enrichment of HPV-16 integrations, cervix, anus, penis and head and neck. By considering CNVs in a region of 1 megabase (Mb) around the HPV integration site, we did not find a significant difference in either the amplification level of the detected CNVs (Fig. S5E) or the absolute number of CNVs (Fig. S5F) between HPV-16 positive and negative samples, althought HPV-negative samples tended to have both higher number and higher amplication of the CNVs.

APOBEC mutation signatures are DNA mutation patterns caused by the APOBEC (Apolipoprotein B mRNA Editing Catalytic Polypeptide-like) family of cytidine deaminases [37], and are characterized by either **C > T or C > G mutations** [38]. HPV has previously been shown to be associated with APOBEC mutation signatures in the host genome [39,40] and certain APOBEC-genes are up-regulated in relation to HPV infection [41]. We aimed to investigate whether these associations persist in the context of metastatic cancer and to identify which APOBEC genes are specifically linked to HPV and if the pattern is consistent in metastatic samples across all HPV-associated cancers. Focusing on cancers with primary tumor location of cervix, anus, penis and head and neck, we found that HPV positive samples had significantly higher expression of several APOBEC3-genes, especially APOBEC3B which was significantly fhigher in anus, penis and head and neck (Fig. 4C). Encouraged by this result we investigated if HPV-positive samples were enriched for APOBEC mutation signatures and found that the APOBEC enrichment score (see Methods) was significantly higher in HPV-positive samples for anus and head and neck and the trend was similar for cervix and penis cancer, although not significant (Fig. 4D). These results indicate that multiple APOBEC-genes are associated with HPV infection and that the association is true for several HPV-associated cancers.

Extrachromosomal DNA is circular DNA that is enriched in tumor tissue [42] and may contain oncogenes that are amplified and highly trancribed [43,44]. HPV has been shown to promote the formation of extrachromosomal DNA (ecDNA), including both host-derived and host-viral ecDNA [[45], [46], [47], [48]]. To explore whether HPV-positive samples exhibit higher ecDNA levels than HPV-negative samples, we focused on host ecDNA and compared the total number of detected host ecDNA between the two groups. We found that HPV-positive samples had a significantly higher number of ecDNA compared to an equal number of randomly selected HPV-negative samples (p = 0.003, one-sided Wilcoxon test) (Fig. 4E). Further, focusing only on the HPV-significant cancers for which ecDNA was detected, cervix, anus and head and neck, we also found a significant higher number of ecDNA in HPV + samples compared to HPV- samples (p = 0.0001 one-sided Wilcoxon test) (Fig. 4F). Next, we investigated if samples with detected host ecDNA had higher levels of HPV compared to samples without host ecDNA. First, when considering all cancer types, we found that ecDNA-positive samples had a significant higher HPV genome coverage compared to ecDNA-negative samples (p = 0.003, two-sided Wilcoxon test) (Fig. 4G). The trend remained the same for beforementioned HPV-associated cancers (Fig. 4H).

### 2.3 The blood viral landscape

**Fig. 5:**
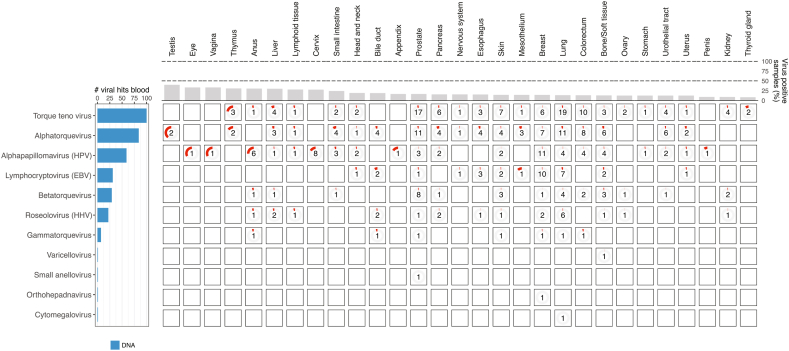
**Overview of virus genera in blood.** Similar as in Fig. 2) for blood samples.

Several viral taxa, such as HPV, EBV, HHV and Anellovirus, are known to be present in blood. Virus in blood may reflect a diseased state, but many viruses, including the abovementioned are also detected in the healthy human virome and not necessarily a sign of ongoing infection or cancer [[49], [50], [51], [52], [53], [54], [55], [56], [57]]. Having analyzed the tissue data, we investigated whether viruses were also present in the matched blood samples and how they distribute between cancer types. We detected 11 unique genera, four of which belong to the Anelloviridae family, with alphatorquevirus as the most prevalent(Fig. 5 and Fig. S1D). HPV was detected in several cancer types, with anus and cervix being the cancer types with highest prevalence relative to sample size (Fig. 5), and with an average genome coverage per sample of 42 % and 48 %, respectively (Fig. S4F). Both cervix and anus cases had a higher proportion of HPV-positive blood samples than expected by chance (Chi-squared test, p < 2.2e-16 and p = 6.7e-12 for Cervix and Anus, respectively). Interestingly, 20 % (n = 8) of the cervix samples and 40 % (n = 6) of the anus samples that were HPV-positive in tissue also had HPV-positive blood samples, indicating that HPV-containing tumor cells can be detected in bloodstream (Fig. S4G).

## 3 Discussion

Here we characterize the tissue and blood virome of 4918 patients with metastatic cancer from the HMF cohort. The virome in metastatic tissue was largely dominated by HPV, EBV and HHV, however, several other genera with lower prevalence were detected. Given the well-established oncogenic roles of HPV and EBV in primary cancers in other pan-cancer datasets such as TCGA [6] and PCAWG [5], it is not unexpected that these viruses remain prevalent in metastatic cancers. However, the extent to which the metastatic virome mirrors that of primary tumors has remained largely unexplored due to lack of deep profiled metastatic tumor samples. Our findings confirm that the viral landscape in metastatic cancer is largely consistent with primary tumors, aligning with previous pan-cancer studies, but also reveal notable variations, particularly in the context of HPV in CRC and the immune response to EBV in metastases.

In line with the known etiology of the virus, HPV was enriched in cancers of the reproductive organs, in addition to head and neck and anus. The relatively high and statistically significant prevalence of HPV in CRC has not previously been reported in large pan-cancer datasets [5,6]. Although the presence of HPV within CRC remains controversial [15,17,[58], [59], [60], [61]], our strict filtering based coverage analysis that reduces false positive detections, supports that HPV is indeed present in colorectal cancer metastases. Our analyses show that HPV + CRC- derived metastases are found in organs like the liver, lung and lymph nodes, body sites that merely do not reflect trophism of HPV but rather the cancer organotrophism, supporting that HPV is brought to the tissue by the metastasizing cells. Furthermore, the RNA-seq data support the active transcription of HPV, suggesting an ongoing HPV infection. To our knowledge the implications of HPV + cells at such body sites is presently unknown and opens new research questions.

Other associations worth noting includes the significant enrichment of HBV in liver cancer, confirming the presence of hepatitis B virus in liver cancer [62]. Of the six HBV positive samples, four were detected in biopsies also located in the liver, whereas two were located in the lung metastases.

Having shown that HPV were significantly associated with several cancer types, we utilized the matched RNA-seq data to investigate potential effects of HPV on metastatic cell host gene expression. The analysis supported previously known associations between HPV and DNA repair processes [[63], [64], [65]]. Samples with HPV showed significant changes in host gene expression compared to HPV-negative samples.

We confirmed the previously known mutual exclusive association between TP53 mutation and HPV in cervix- and head and neck cancer [5], and further showed that this association is also true for anus and penis cancer. However, we were unable to validate previously reported mutually exclusive associations between head-and-neck HPV-positivity and the genes CDKN2A, TERT, and FAT15 [5]. This could be due to too few cases of HPV-positive head-and-neck cancers or differences in exclusivity between primary and metastatic samples. We also confirm a previous finding from the PCAWG cohort that HPV-positive samples have higher mutational load compared to HPV-negative samples [5]. Extrachromosomal DNA has emerged as a key player in cancer biology, particularly in its role in oncogene amplification and tumorigenesis [[66], [67], [68]]. Recent studies have highlighted the involvement of host-virus ecDNA, where viral genomes, such as those from HPV, exist in extrachromosomal circular forms within host cells [45,46]. We focused the analysis on host ecDNA, and showed for the first time to our knowledge using a pan-cancer WGS dataset, that HPV-positive samples have more host ecDNA compared to HPV-negative samples. This association could be caused by the increased genome instability we observed in HPV-positive samples. Since our analysis focused only on host-derived ecDNA, a more comprehensive approach that also includes viral-host ecDNA could provide more insights into the role of HPV within cancer cells.

While HPV is well established to affect host genome stability, other viruses could also cause genome instability and affect host cellular processes, including for instance Human cytomegalovirus (HCMV) [69] EBV [70] and HBV [71]. Future analyses of these and other less prevalent viruses with respect to the mutational and transcriptional landscape of the host genome, could reveal new mechanisms for virus-induced oncogenesis or novel virus-cancer associations.

Leveraging matched RNA-seq with CIBERSORTx to identify infiltrating immune cells we identified significant correlations between EBV and CD8^+^ T-cells and EBV and plasma cells, in agreement with previous studies [19,20]. EBV latency is maintained in resting B lymphocytes, while the lytic replication cycle is activated when B cells differentiate into plasma cells [72]. CD8^+^ T cells are the primary responders to EBV and restrict expansion of infected cells with both latent and lytic EBV infection. CD8^+^ T cells will be rapidly expanding in response to EBV and is recruited to sites of infection [73,74]. CD8^+^ T cells are also the most powerful effector cells in the anti-tumor immune response. The increased presence of immune cells within EBV-positive tumors has been shown to increase responsiveness to immunotherapy, but EBV specific T cells may also be exhausted and there may be need for alternative therapeutic strategies to overcome the dysfunctional state [75,76].

Identifying cancer type-specific virus associations could have important implications in various aspects of cancer detection and treatment. Cancer of unknown primary (CUP) includes about 5 % of cancers and is associated with increased mortality compared to cancers with known primary [77]. Using cancer type-specific viruses to help identify the primary lesion of a metastatic biopsy could help diagnose certain CUPs. We demonstrate that HPV in metastases alone could serve as a valuable biomarker for identifying CUPs originating from the cervix, penis, anus, and vagina. Although these cancers represent only a small fraction of diagnosed cases, HPV could be a useful biomarker for screening squamous cell carcinoma in inguinal lymph nodes of unknown primary origin, potentially aiding in identifying these cancer types. If WGS of tumor biopsies becomes a routine diagnostic practice, HPV screening would be easy to implement.

Extending HPV-screening to a blood-based test would further ease the detection and complement the tissue samples. HPV in blood has been investigated due to its potential as a diagnostic biomarker for HPV-related cancers such as oral squamous cell carcinoma and cervical cancer, including both HPV-antibodies and PCR-based DNA-tests [78,79]. In our data, head and neck, cervix and anus had the highest mean genome coverage of HPV in blood, although it was not consistent for all samples. We detected HPV in a significant number of anus and cervix samples, pointing towards a potential blood-based biomarker signal for these cancer types. Since HPV is also detected in blood of other cancer types, a tumor specific biomarker based on HPV would need to be refined using additional filters such as higher genome coverage. Apart from HPV, the blood samples were mainly dominated by viruses of the *Anelloviridae* family. *Anelloviridae* is common in blood and has multiple routes of transmission and appears to be person-specific [80] and genetically diverse [57], and although some studies have indicated an association between anelloviruses and certain diseases, they are in general believed not to be directly related to human disease [11,81].

Contamination is a critical concern in low-biomass samples, such as human tissue and blood samples. Recent discussions have raised awareness about the issue of contamination when analyzing low-biomass samples using data not optimized for detecting microbes [82].

By using a coverage based detection approach of about 30 million host-depleted reads, we successfully showed that virus are detectable in metastatic tissue and blood. We showed that the viral reads are distributed across most of the viral genomes, indicating that the viral sequences are not due to external contamination from a single vector or PCR-product. Furthermore, the most prevalent viruses are detected in both the DNA and RNA data, indicating that the viruses are present and actively transcribed in the host-cells. Further, we only considered viruses that are previously shown to be associated with humans. We therefore assume that our viral hits as true associations, although external contamination cannot be ruled out completely. Further studies optimized for microbial detection, with paired control samples, microbial enrichment steps, and using multiple methodologies, will be important to fully elucidate the viral landscape in cancer.

## 4 Material and methods

### 4.1 Hartwig Medical Foundation (HMF) cohort

The HMF cohort ^9^contained patient data provided under data transfer agreement (DR-222) by Hartwig Medical Foundation. The HMF data comprises WGS data from blood and tissue and RNA-seq data from tissue. The clinical data consists of primary tumor type, biopsy location, gender, age, pretreatment type, date of sample collection and date of death. Biopsy site was unknown for 1014 samples; 2892 samples were from distant metastasis to other organs; 786 samples were from lymph node metastasis and 260 samples were from the primary tumor. Normal samples (blood) had a mean read coverage of ∼38x while tumor samples had a mean coverage of ∼106x [9].

### 4.2 Identifying and removing contaminants

For the detection of virus we applied a coverage-based filtering procedure, requiring a per-samples genome-coverage of at least 10 % for a virus to be regarded as present in a sample. In addition, we removed the taxa simplexvirus, which has previously been identified as a viral contaminant [5] and we removed all retroviruses (human endogenous retrovirus, gammaretrovirus, betaretrovirus and deltaretrovirus) and the taxa lentivirus. Further, some viruses with known human associations were detected in only one sample although with a genome-coverage above 10 %, including alphainfluenzavirus, betacoronavirus, betatorquevirus, bocaparvovirus, norovirus, orthopneumovirus, pneumoviridae and protoparvovirus*.* Those were regarded as detected. Finally, for the top three most abundant viruses (*Alphapapillomavirus, Lymphocryptovirus* and *Roseolovirus*) we investigated the genome-coverage plots across all cancer types to look for potential contaminant patterns, as previously described by Dohlman et al. [83]. For these three taxa, we found no signs of biological or technical contamination: The taxa showed different coverage and read-depth across cancer types, with increased abundance in cancer types for which the virus is expected to be present.

### 4.3 Preparation of data

Whole-genome sequencing data were made available from HMF via Google Cloud Platform as aligned cram-files. Unmapped human reads were extracted from the cram files using *samtools view* with the parameter *-f4*. The human-read depleted output files were filtered by length and Illumina adapters using *trim_galore* with the following parameters *--illumina --stringency 5 --length 75 --max_n 2 --trim-n -j 5*. The output files were used for microbial profiling. The RNA-seq data was made available as raw fastq-files. The forward and reverse reads were aligned to the human genome (hg19) using STAR-2.7.9a with the following parameters: *-outSJfilterReads Unique --outSJtype None --outFilterIntronStrands RemoveInconsistentStrands --outFilterIntronMotifs RemoveNoncanonicalUnannotated --outFilterScoreMin 10 --outFilterMismatchNmax 5 --outSAMtype BAM Unsorted --outReadsUnmapped Fastx --outSAMunmapped None --outSAMmultNmax 1 --readFilesCommand gunzip --readFilesIn R1.fastq.gz R2.fastq.gz*. A forward and reverse file with unmapped human reads were produced by STAR which were used for microbial profiling.

### 4.4 Messenger-RNA and mutation data from HMF

The gene-expression data was made available from HMF and is the output from the HMF-Isofox pipeline (https://github.com/hartwigmedical/hmftools/tree/master/isofox). The driver-mutation data was also made available from HMF. The output files ∗linx.driver.catalog.tsv from the Linx pipeline were used for the mutation analyses (https://github.com/hartwigmedical/hmftools/tree/master/linx).

### 4.5 Differential expression analyses

Differential expressed genes between HPV-groups were determined using *limma-voom (v3.52)* in R. Data was normalized using TMM. For the comparison between HPV+ and HPV- samples, we required genes to be expressed with at least 1 count in 50 % of the samples. ENSEMBL IDs were converted to Gene-symbols using the R-package *org.Hs.eg.db.* GO-analysis for genes differentially expressed between HPV+ and HPV- samples were performed using the R package *clusterProfiler* (v.4.4.4) using the function enrichGO with parameters *ont= "BP",pAdjustMethod = "BH",pvalueCutoff = 0.01, qvalueCutoff = 0.01*. Expressed genes in the dataset were used as background (“universe”). For the GO-analysis, up-regulated genes were defined as genes with logFC>0 and Benjamini-Hochberg adjusted p-values <0.01. Down-regulated were defined as genes with logFC<0 and Benjamini-Hochberg adjusted p-values <0.01.

### 4.6 HPV classification

To evaluate HPV's potential as a diagnostic marker for primary tumor location, we performed receiver operating characteristic (ROC) curve analysis. For each cancer type, we conducted one-versus-all comparisons using two approaches: 1) a binary classification where samples were considered HPV-positive if their HPV coverage exceeded 10 %, and 2) using the continuous HPV coverage values directly. Area Under the Curve (AUC) values were calculated using the pROC package (version 1.18.5) in R. This analysis was performed separately for each primary tumor location against all other locations to assess HPV's ability to distinguish specific cancer types. AUC values range from 0 to 1, where 1 indicates perfect classification and 0.5 represents random chance. Additionally, we performed a focused analysis on HPV-associated cancers (cervical, penile, anal, and vaginal) to assess HPV's ability to discriminate between these specific cancer types. For each cancer type, we calculated descriptive statistics including mean and median HPV coverage, total sample count, and number of HPV-positive cases (defined as >10 % coverage). ROC curves were generated comparing each HPV-associated cancer type against the others based on HPV coverage values, producing ROC curves and corresponding AUC values for each cancer type. Results were visualized using ggplot2, comparing the performance of binary and continuous classification approaches across cancer types, with ROC curves plotted against a diagonal reference line representing random classification (AUC = 0.5).

### 4.7 Extrachromosomal DNA (ecDNA) analysis

The ecDNA data was was provided by HMF by their linx-mutation calling pipeline (https://github.com/hartwigmedical/hmftools). For each sample, the total number of ecDNA was determined by summing the number of detected ecDNA. An ecDNA-positive sample was defined as a sample having at least one detected ecDNA.

### 4.8 Correlating sequencing read number with number of viral hits

To investigate the effect of reducing the number of sequencing reads on the pipeline's ability to detect viruses, we compared the number of viral hits using the full dataset of unmapped host-reads (all available unmapped host-reads) with results from 75 % to 50 % randomly selected unmapped host reads from the full set of unmapped reads. For computational purposes, this re-analysis was conducted on a subset of 2000 randomly selected WGS samples.

### 4.9 Virus analysis

RefSeq viral genomes were downloaded from NCBI by selecting human hosts taxa as defined by NCBI, which included 840 unique viruses (Supplementary Table 2). The WGS-data was aligned to the RefSeq viral genomes using *Bowtie2 (v2.3.4.1)*. A pileup of the aligned reads was created using *samtools (v1.7) mpileup*. Genome coverage was calculated from the aligned reads using *bedtools (v2.26.0) genomecov.* The RNA data were analyzed similarly as the DNA-data using *Bowtie2*. Viruses with a genome coverage per sample above 10 % were regarded as detected. Summed genome coverage plots were created by calculating the sum of the coverage for all samples per genome position.

Viral integrations were detected by VIRUSBreakend from the GRIDSS module [84] (gridsstools-src-2.12.2) using the database *virusbreakenddb_20210401* (https://github.com/PapenfussLab/gridss) and the hg19 reference genome provided by HMF (https://github.com/hartwigmedical/hmftools) using the parameter *--minviralcoverage 1.* Additional dependencies include *bcftools-1.14* and *samtools-1.15.1*.

APOBEC signatures were characterized using the function *trinucleotideMatrix* within the R-package *MutationalPatterns* (v3.16.0).

Associations between viruses and immune cell composition were investigated by CIBERSORTx (https://cibersortx.stanford.edu) using TPM-normalized RNA-seq data as input, made available from HMF. The CIBERSORTx absolute score was compared between groups.

Association between TP53 and HPV was calculated using Fisher's exact test in R by comparing HPV-status and TP53-mutation status. The p-values were adjusted for multiple testing across all cancer types using the function *qvalue* in R. The mutation data was provided by HMF and generated by their mutation calling pipeline (https://github.com/hartwigmedical/hmftools). Single nucleotide variation (SNV)-mutation load between HPV16 + and HPV16 - samples was calculated by 1) defining a region of 10 kb upstream and downstream of HPV integration as identified by VIRUSBreakend; 2) intersect the virus integration site with gene coordinates to define the gene body; 3) summing the number of single-nucleotide variants within the 20 kb region per sample; 4) sum the number of single-nucleotide variants within the same 20 kb genomic region for a random set of samples without HPV-integration. The correlation between copy number variants (CNV) and HPV was conducted in a similar way as for SNVs, expanding the region to 1 Mb around the integration site.

Correlation between host gene expression and HPV16-integration was determined by 1) identify HPV16+ samples for the four cancer types that were included in the analysis; 2) identify genes with HPV16+ integrations for the selected samples; 3) determine the gene expression of the genes identified in step 2; 4) determine the gene-expression for the same genes as identified in step 2, for samples within the same four cancer types that did not have HPV16 integration.

## CRediT authorship contribution statement

**Robin Mjelle:** Writing – review & editing, Writing – original draft, Supervision, Investigation, Formal analysis, Conceptualization. **Ícaro Castro:** Writing – review & editing, Writing – original draft, Formal analysis. **Kristin Roseth Aass:** Writing – review & editing, Writing – original draft, Formal analysis.

## Consent to publish

None.

## Consent to participate

None.

## Data availability

The raw data is available upon application to Hartwig Medical Foundation https://www.hartwigmedicalfoundation.nl/en/

## Ethical approval

This study reanalyzes data from the Hartwig Medical Foundation, which was collected in accordance with ethical regulations.

## Funding

This study was supported by a grant from The Joint Research Committee between St. Olavs hospital and the 10.13039/100019902Faculty of Medicine and Health Sciences, NTNU (FFU) (Grant# 46055600–113 and 46055600–134) and from the post-doc grant from The Liaison Committee for Education, Research and Innovation in Central Norway (Samarbeidsorganet) (Grant# 2020/39645).

## Declaration of competing interest

The authors declare that they have no known competing financial interests or personal relationships that could have appeared to influence the work reported in this paper.
