# Supplementary figures and images for: The viral landscape in metastatic solid cancers
Source: Heliyon. 2025 Feb 8;11(4):e42548. doi: 10.1016/j.heliyon.2025.e42548 (PMC11870251; doi:10.1016/j.heliyon.2025.e42548)

**a**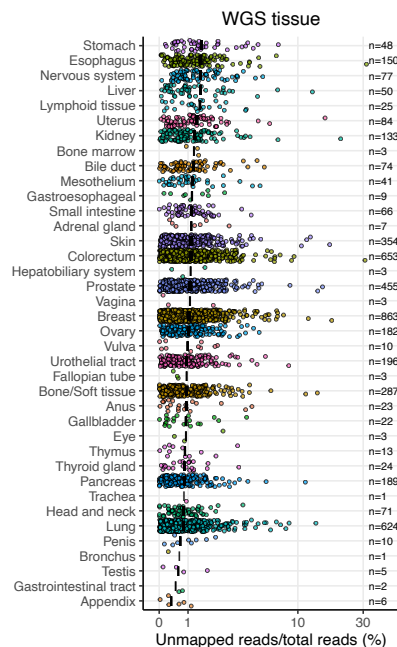**b**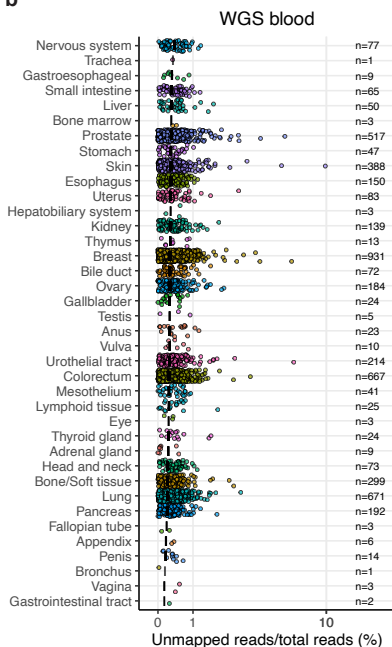**c**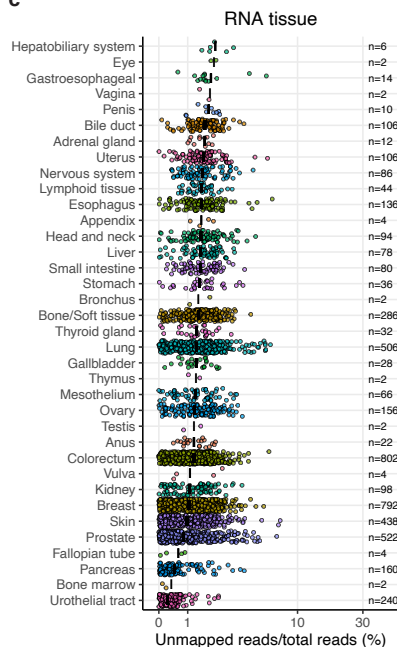**d**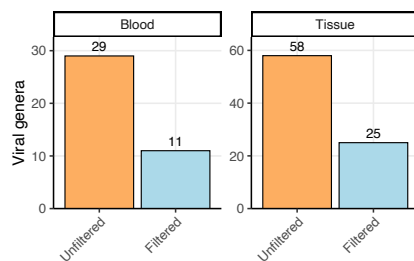

Supplement: Multimedia component 3 [file mmc3.pdf]

**a**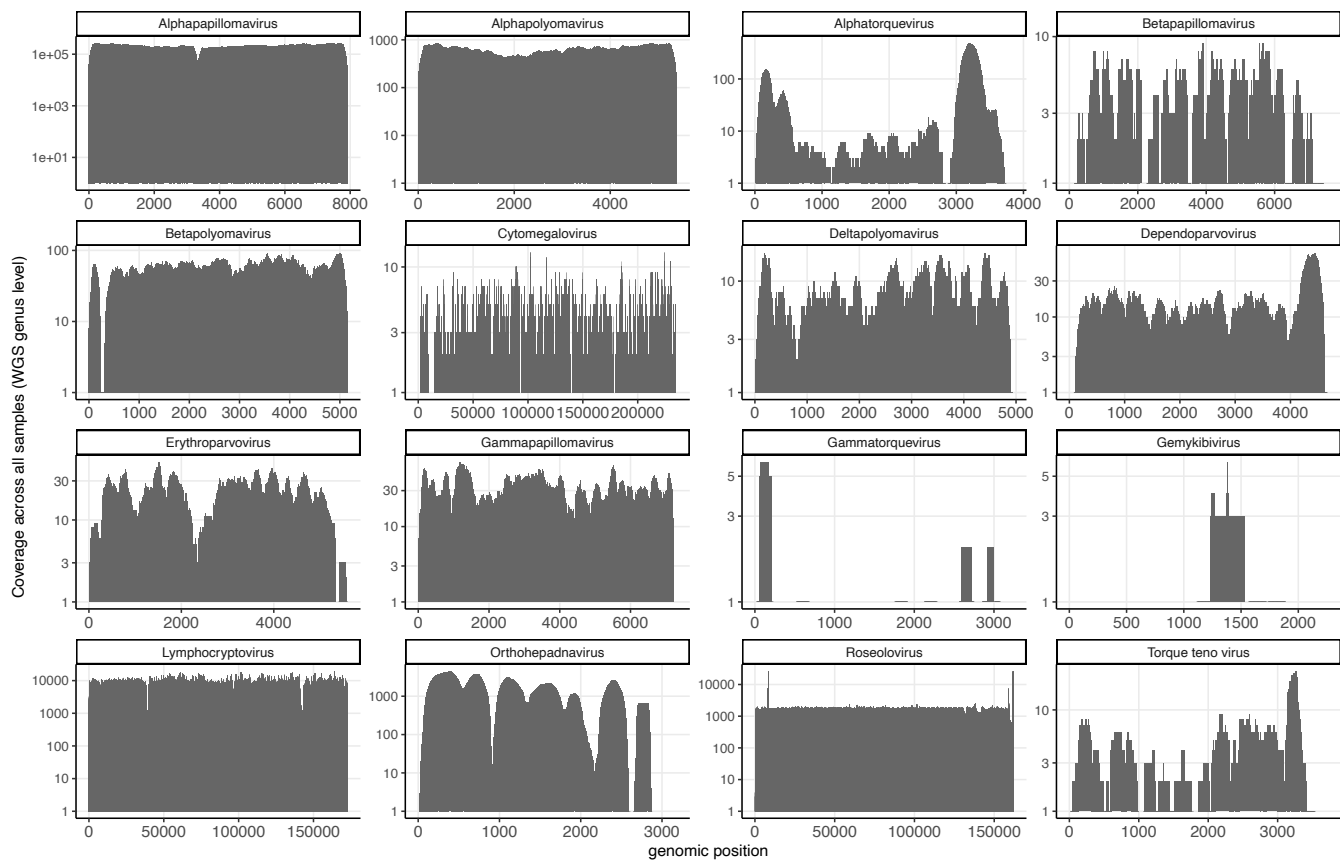**b**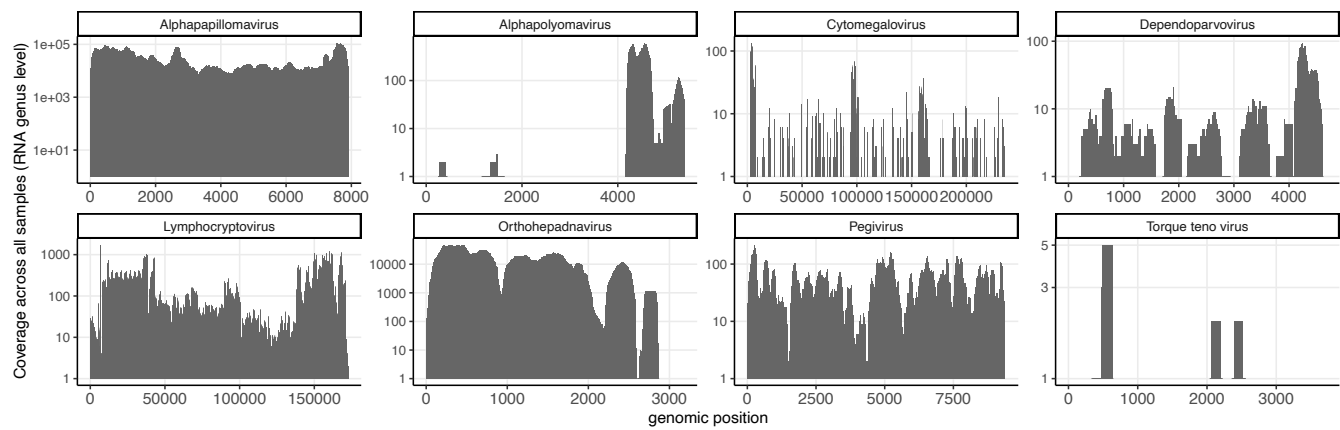**c**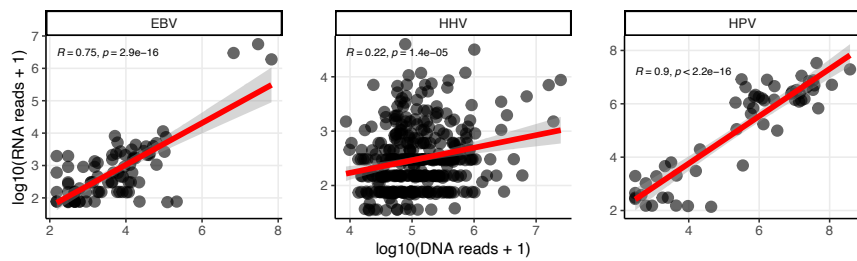**d**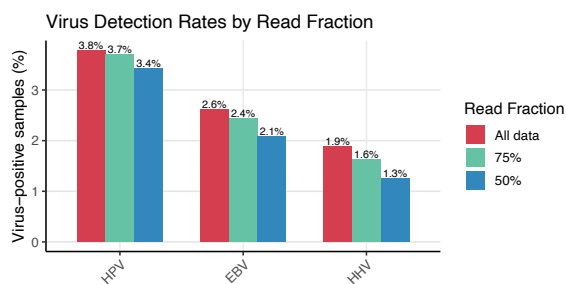

Supplement: Multimedia component 4 [file mmc4.pdf]

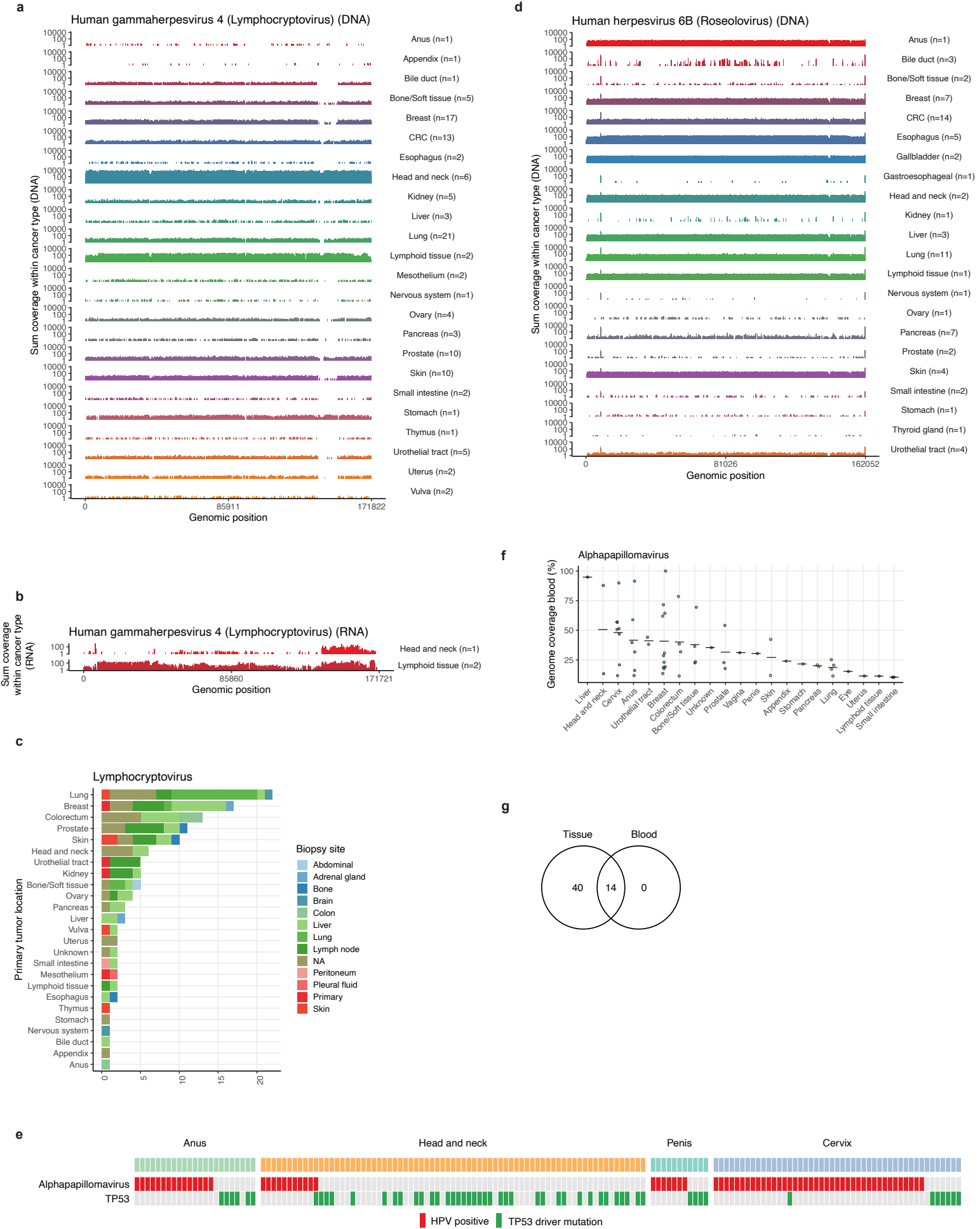

Supplement: Multimedia component 6 [file mmc6.pdf]

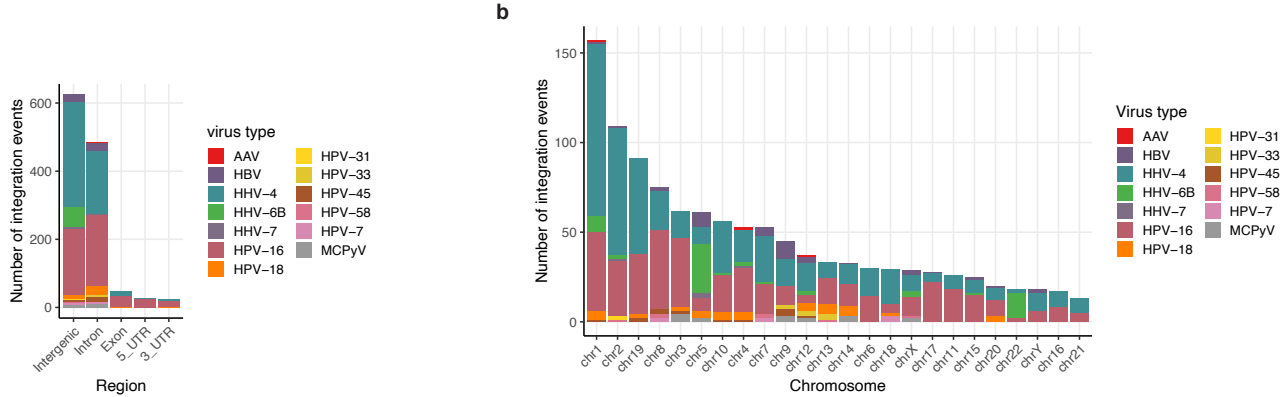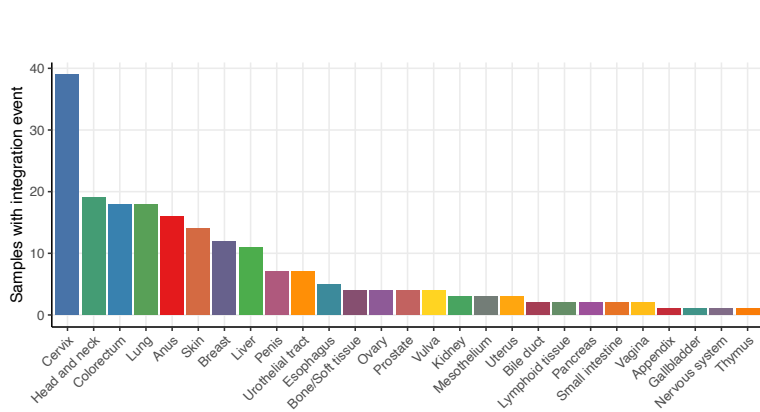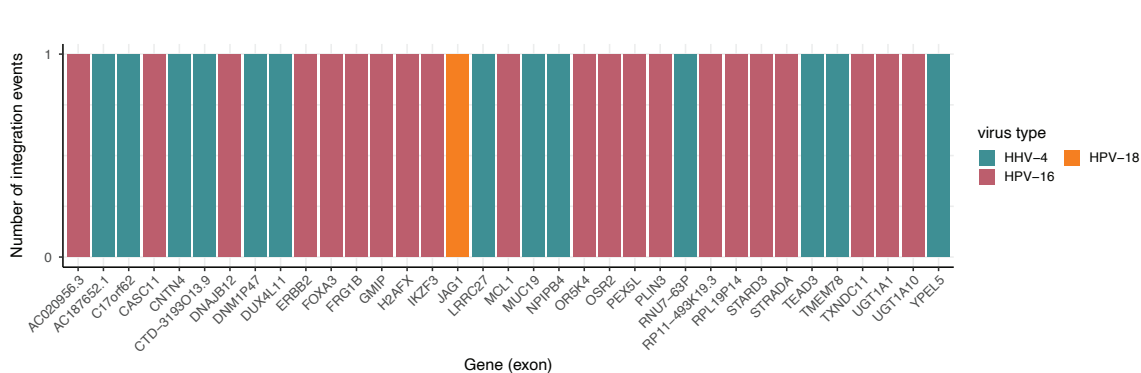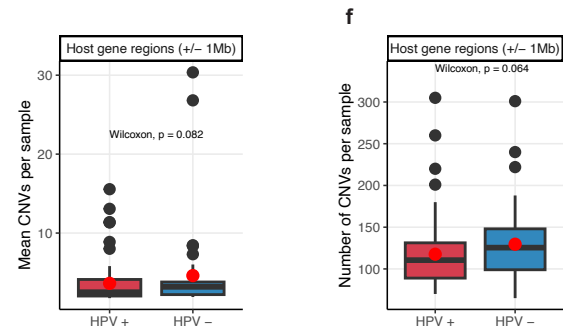

Supplement: Multimedia component 7 [file mmc7.pdf]
