# Supplementary material for: The viral landscape in metastatic solid cancers
Source: Heliyon. 2025 Feb 8;11(4):e42548. doi: 10.1016/j.heliyon.2025.e42548 (PMC11870251; doi:10.1016/j.heliyon.2025.e42548)
Supplement: Multimedia component 5 [file mmc5.pdf]

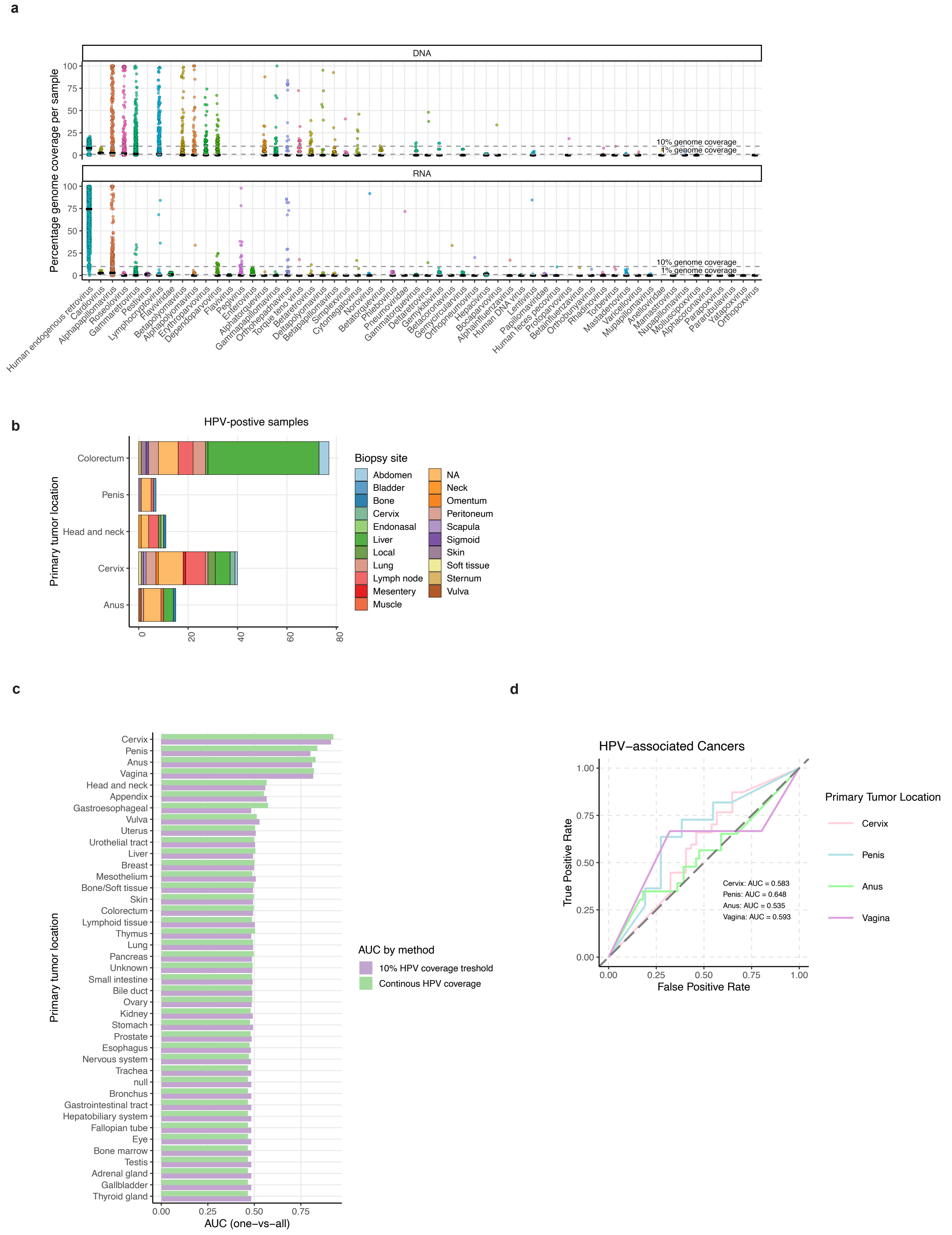
a
DNA
RNA
Percentage genome coverage per sample
100
75
50
25
0
10% genome coverage
1% genome coverage
Human endogenous retrovirus
Cardiovirus
Alphapapillomavirus
Roseolovirus
Gammaretrovirus
Pestivirus
Lymphocryptovirus
Flaviviridae
Betapolyomavirus
Alphapolyomavirus
Erythroparvovirus
Dependoparvovirus
Flavivirus
Pegivirus
Enterovirus
Alphatorquevirus
Gammapapillomavirus
Orthohepadnavirus
Torque teno virus
Betaretrovirus
Deltapolyomavirus
Betapapillomavirus
Simplexvirus
Cytomegalovirus
Norovirus
Betatorquevirus
Phlebovirus
Pneumoviridae
Gammatorquevirus
Deltaretrovirus
Gemykibivirus
Betacoronavirus
Gemycircularvirus
Orthopneumovirus
Hepacivirus
Bocaparvovirus
Alphainfluenzavirus
Human DNA virus
Lentivirus
Papillomaviridae
Human feces pecovirus
Protoparvovirus
Betainfluenzavirus
Orthobunyavirus
Rhadinovirus
Torbevirus
Mastadenovirus
Varicellovirus
Mupapillomavirus
Anelloviridae
Mamastrovirus
Nupapillomavirus
Molluscipoxvirus
Alphacoronavirus
Parapoxvirus
Pararubulavirus
Yatapoxvirus
Orthopoxvirus
b
HPV-postive samples
Primary tumor location
Colorectum
Penis
Head and neck
Cervix
Anus
0
20
40
60
80
Biopsy site
Abdomen
Bladder
Bone
Cervix
Endonasal
Liver
Local
Lung
Lymph node
Mesentery
Muscle
NA
Neck
Omentum
Peritoneum
Scapula
Sigmoid
Skin
Soft tissue
Sternum
Vulva
c
Primary tumor location
Cervix
Penis
Anus
Vagina
Head and neck
Appendix
Gastroesophageal
Vulva
Uterus
Urothelial tract
Liver
Breast
Mesothelium
Bone/Soft tissue
Skin
Colorectum
Lymphoid tissue
Thymus
Lung
Pancreas
Unknown
Small intestine
Bile duct
Ovary
Kidney
Stomach
Prostate
Esophagus
Nervous system
Trachea
null
Bronchus
Gastrointestinal tract
Hepatobiliary system
Fallopian tube
Eye
Bone marrow
Testis
Adrenal gland
Gallbladder
Thyroid gland
0.00
0.25
0.50
0.75
AUC (one−vs−all)
AUC by method
10% HPV coverage treshold
Continous HPV coverage
d
HPV−associated Cancers
True Positive Rate
False Positive Rate
1.00
0.75
0.50
0.25
0.00
0.00
0.25
0.50
0.75
1.00
Cervix: AUC = 0.583
Penis: AUC = 0.648
Anus: AUC = 0.535
Vagina: AUC = 0.593
Primary Tumor Location
Cervix
Penis
Anus
Vagina
